# Communicating uncertainties when disclosing diagnostic test results for (Alzheimer's) dementia in the memory clinic: The ABIDE project

**DOI:** 10.1111/hex.12964

**Published:** 2019-10-22

**Authors:** Leonie N. C. Visser, Sophie A. R. Pelt, Marleen Kunneman, Femke H. Bouwman, Jules J. Claus, Kees J. Kalisvaart, Liesbeth Hempenius, Marlijn H. de Beer, Gerwin Roks, Leo Boelaarts, Mariska Kleijer, Wiesje M. van der Flier, Ellen M. A. Smets, Marij A. Hillen

**Affiliations:** ^1^ Department of Medical Psychology Amsterdam Public Health Research Institute University of Amsterdam Amsterdam UMC Amsterdam The Netherlands; ^2^ Department of Neurology Alzheimer Center Amsterdam Amsterdam Neuroscience Vrije Universiteit Amsterdam Amsterdam UMC Amsterdam The Netherlands; ^3^ Knowledge and Evaluation Research (KER) Unit Mayo Clinic Rochester MN USA; ^4^ Department of Biomedical Data Sciences Medical Decision Making Leiden University Medical Center Leiden The Netherlands; ^5^ Department of Neurology Tergooi Hospital Blaricum The Netherlands; ^6^ Department of Clinical Geriatrics Spaarne Gasthuis Haarlem The Netherlands; ^7^ Geriatric Center Medical Center Leeuwarden Leeuwarden The Netherlands; ^8^ Department of Neurology Reinier de Graaf Gasthuis Delft The Netherlands; ^9^ Department of Neurology ETZ Hospital Tilburg The Netherlands; ^10^ Geriatric Department NoordWest Ziekenhuis Groep Alkmaar The Netherlands; ^11^ Department of Neurology LangeLand Ziekenhuis Zoetermeer The Netherlands; ^12^ Department of Epidemiology and Biostatistics Vrije Universiteit Amsterdam Amsterdam UMC Amsterdam The Netherlands

**Keywords:** Alzheimer's disease, dementia, diagnostic work‐up, memory clinic, physician‐patient communication, uncertainty

## Abstract

**Background:**

The development of novel diagnostics enables increasingly earlier diagnosis of Alzheimer's disease (AD). Timely diagnosis may benefit patients by reducing their uncertainty regarding the cause of symptoms, yet does not always provide patients with the desired certainty.

**Objective:**

To examine, using both quantitative and qualitative methods, uncertainty communicated by memory clinic clinicians in post‐diagnostic testing consultations with patients and their caregivers.

**Methods:**

First, we identified all uncertainty expressions of 22 clinicians in audiotaped post‐diagnostic testing consultations with 78 patients. Second, we statistically explored relationships between patient/clinician characteristics and uncertainty expressions. Third, the transcribed uncertainty expressions were qualitatively analysed, determining the topic to which they pertained, their source and initiator/elicitor (clinicians/patients/caregivers).

**Results:**

Within 57/78 (73%) consultations, clinicians expressed in total 115 uncertainties, of which 37% elicited by the patient or caregiver. No apparent relationships were found between patient/clinician characteristics and whether or not, and how often clinicians expressed uncertainty. Uncertainty expressions pertained to ten different topics, most frequently patient's diagnosis and symptom progression. Expressed uncertainty was mostly related to the unpredictability of the future and limits to available knowledge.

**Discussion and conclusions:**

The majority of clinicians openly discussed the limits of scientific knowledge and diagnostic testing with patients and caregivers in the dementia context. Noticeably, clinicians did not discuss uncertainty in about one quarter of consultations. More evidence is needed on the beneficial and/or harmful effects on patients of discussing uncertainty with them. This knowledge can be used to support clinicians to optimally convey uncertainty and facilitate patients' uncertainty management.

## 1 INTRODUCTION

About 50 million people are currently living with dementia worldwide, and this number is rapidly increasing due to the ageing population.^1^ Alzheimer's disease (AD) is the most common cause of dementia, with no existing cure yet.^2^ Many aspects of this disease are still unknown. Clinical progression is, for example, difficult to predict, impairing patients' and their caregivers' ability to plan ahead and prepare for the future. Although uncertainty has always been inherent to the AD and dementia context, two recent developments have expanded the scope of uncertainty with which patients, caregivers and clinicians are confronted. First, additional uncertainty has resulted from the introduction of ‘pre‐dementia’ diagnostic categories in memory clinic practice, such as ‘mild cognitive impairment (MCI)’.^3^, ^4^ Roughly half of MCI patients develop dementia in the course of 3 years, while the other half remains stable or improves.^5^ Whether, when and if so how MCI patients will develop dementia is still difficult to predict.^6^, ^7^ Second, novel diagnostic measures are developed to enable earlier and a more accurate diagnosis of AD.^8^, ^9^ Examples are tests detecting biomarkers through imaging techniques or in cerebrospinal fluid (CSF).^10^, ^11^ Earlier diagnostic testing might benefit some patients and their caregivers by reducing uncertainty regarding the cause and the course of the patient's symptoms.^12^, ^13^, ^14^, ^15^, ^16^ However, these tests do not always provide the desired certainty.^17^ The interpretation of diagnostic test results is often complicated, for instance when they are borderline abnormal or conflicting.^9^ In addition, for individuals without dementia, results of AD‐biomarker diagnostic tests only yield a risk indication of developing dementia within the next years.^13^ Concluding, the introduction of both ‘MCI’ as a diagnostic category and (early) biomarker testing, may have heightened uncertainty for patients and caregivers in the diagnostic trajectory for (AD) dementia.

The phenomenon of uncertainty in illness can be described as someone's ‘subjective consciousness or awareness of one's lack of knowledge’.^18^, ^19^, ^20^, ^21^, ^22^ Uncertainty may relate to a wide variety of topics, varying from disease progression to test reliability. In addition, several distinct types or ‘sources’ of uncertainty can be distinguished,^18^ that is (a) probability, (b) ambiguity and (c) complexity. ‘Probability’ refers to uncertainty caused by an inability to predict the future. Probability most frequently takes on the form of a risk, such as a 50% chance to develop dementia after an MCI diagnosis. ‘Ambiguity’ refers to uncertainty resulting from the lack of reliable, credible or adequate information about a phenomenon. An example is the uncertainty resulting from conflicting test results. Finally, ‘complexity’ refers to uncertainty due to difficulties in comprehending aspects of the phenomenon itself—for example uncertainty about a definitive diagnosis resulting from the interplay between a multitude of complex factors.^18^ Besides conceptual work on uncertainty, previous research so far has focused on how clinicians cope with uncertainty. Evidence outside of the context of dementia suggests that clinicians vary in their recognition and acknowledgement of uncertainty, and are often hesitant to discuss uncertainty with patients.^23^, ^24^, ^25^, ^26^, ^27^ A recent observational study in the field of cancer genetic counselling indicates that clinicians do express and address uncertainty, but to a widely varying degree.^28^ Several factors may explain such variation, first of which is clinicians' general tendency in responding to uncertainty—their tolerance for uncertainty.^29^ Clinicians' tolerance for uncertainty has been suggested to influence their openness in sharing uncertainty with patients. Second, clinicians may adapt the amount of uncertainty they convey depending on patient characteristics such as education level or diagnosis. Evidence supporting these hypotheses is, however, scarce.^30^, ^31^

We do not know yet to what extent and how clinicians in the (AD) dementia diagnostic context discuss uncertainty with patients and caregivers, or which characteristics predict uncertainty communication. Communicating uncertainty is challenging, but often cannot be avoided, especially when patients and/or caregivers are actively involved in the medical interaction by asking questions. To enable the development of strategies for clinicians to optimally address uncertainty and support patients' and caregivers' coping with uncertainty,^32^ we need to first establish the spectrum of uncertainties communicated by clinicians within this context.

In this study, we aimed to examine if and how often clinicians communicate uncertainty in disclosure consultations in the memory clinic setting, to what topics the uncertainty relates and what its sources are. We also examined who elicited clinicians' expressions of uncertainty, that is whether clinicians expressed uncertainties on their own initiative or in response to remarks or questions by patients or caregivers. Additionally, we explored whether the observed amount of uncertainty expressions was related to clinician and/or patient characteristics, such as clinicians' tolerance of uncertainty and patients' diagnosis.

## 2 METHODS

### 2.1 Design and study context

In an observational design, we combined qualitative, descriptive and exploratory quantitative analysis of clinician‐patient consultations in a memory clinic setting in which results of diagnostic testing for AD and dementia were discussed. This study was part of a larger research project entitled Alzheimer's Biomarkers in Daily Practice (ABIDE).^9^ ABIDE addresses the value and use of diagnostic tests for AD to daily practice in memory clinics, including communication about test results.^9^ ABIDE encompasses an observational study of clinical encounters at eight Dutch memory clinics.^33^ During the routine diagnostic work‐up for dementia at those clinics, we audiotaped clinician‐patient consultations, that is the clinical encounter(s) prior to and after diagnostic testing. The current study focused on the latter encounters only. The board of the Medical Ethics Committee of the Academic Medical Centre (AMC) Amsterdam reviewed and approved of this study.

### 2.2 Sample and procedure

All clinicians at the eight memory clinics, that is neurologists and geriatricians, were invited to participate in this study. They were eligible if they were involved in patient consultations during the diagnostic process and if they were willing to participate. Their newly referred patients and patients' informal caregivers who accompanied them to the memory clinic were invited to participate prior to their first visit at the memory clinic. Only patients with sufficient comprehension of the Dutch language who were willing and able to sign informed consent were included (based on clinicians' evaluation). In the current analyses, we included fully recorded consultations (June 2016‐July 2017) in the Dutch language.

### 2.3 Measures

Prior to audiotaping their consultations, clinicians completed a questionnaire assessing sociodemographic and work‐related characteristics, that is their age, gender, specialty (neurology or geriatrics) and level of experience (years of experience and estimated number of new patients per month). In addition, clinicians' tolerance of uncertainty was assessed with nine items from the Physicians' Reaction to Uncertainty Scale (PRUS).^34^ We used the subscales ‘Anxiety due to uncertainty’ and ‘Reluctance to disclose uncertainty to patients’, excluding one item on the use of treatment (6‐point Likert scale ranging from 1: strongly disagree to 6: strongly agree). Item scores were summed to a total score, with a higher score indicating lower tolerance for uncertainty (range 9‐54). Patients and their caregivers completed a questionnaire assessing sociodemographic characteristics, that is age, gender, educational level and (only for caregivers) their relation to the patient. Patients also completed an adapted version of the PRUS to assess their tolerance of uncertainty. We used the subscales ‘Anxiety due to uncertainty’, ‘Reluctance to disclose uncertainty to patients’ and one item from the subscale ‘Concern about bad outcomes’ (six‐point Likert scale strongly disagree to strongly agree). Following the approach of Politi et al,^27^ items were parallel to the original scale.^34^ For instance, if the item for clinicians stated ‘I usually feel anxious when I am not sure of a diagnosis’, the wording was adapted to patients to state ‘I usually feel anxious when I am not sure of my diagnosis’. Total scores were calculated by summing the responses on the eleven items, with higher scores indicating less tolerance for uncertainty (range 11‐66). Patients' Mini‐Mental State Examination (MMSE) score was retrieved from their medical records (range 0‐30, with higher scores indicate better cognitive function). Patients were retrospectively categorized into four diagnostic categories based on data retrieved from their medical record (for more details, see^33^): (a) Dementia, (b) MCI, (c) Cognitively normal or (d) Other/Unclear; that is, another neurological disease was diagnosed, or diagnosis was postponed or unclear.

### 2.4 Analyses

Two trained research assistants (BA, AH) listened to all consultations twice and marked any instances in which they perceived the clinician to express uncertainty. They interpreted the content of the conversation and marked all verbal expressions reflecting the clinician's awareness of uncertainty or a lack of certainty (eg, ‘that's uncertain’ or ‘I am not sure about…’), a chance, risk or probability (eg, ‘there is a chance that…’ or ‘the risk of developing dementia is 50%’), or missing, ambiguous or indefinite knowledge or information (eg, ‘we do not know that yet’, ‘that could still mean two things’, or ‘we don't know what will happen in the future’).^18^ An ‘expression’ could be one or several sentences long. An expression was considered finished when the focus was no longer on uncertainty. All consultations in which *no* uncertainty expressions had been marked were double‐checked by the first author (SP) for uncertainty expressions. If in doubt, BA, AH and SP were instructed to mark the expression, so that it could be checked for inclusion by MH, an expert on uncertainty communication. Next, all sentences in which uncertainty expressions were identified were transcribed verbatim. We also transcribed the clinician‐patient interaction immediately preceding the uncertainty expression (all sentences necessary to understand the uncertainty expression and the initiation of this topic).

We used descriptive statistics to report characteristics of clinicians and patients/caregivers and to report how often clinicians expressed uncertainty. To explore relationships between participant characteristics and uncertainty expressions (ie, the number of expressions and a dichotomous variable indicating if uncertainties were expressed ‘yes’ or ‘no’), we used correlations, *t* tests, Kruskal‐Wallis and Mann‐Whitney *U* tests, and the Chi‐square statistic, depending on the normality and type of data. Significance testing was done two‐sided at an alpha of .05. All quantitative analyses were performed using IBM SPSS Statistics 24.

Next, MAXQDA 12 software^35^ was used to assist in further organizing and analysing the transcribed uncertainty expressions. All transcripts were independently read and double coded, that is by SP and MH or LV, who have backgrounds in psychology and are trained in qualitative data analysis. Codes were subsequently compared and discussed until consensus was reached. First, we identified to which topic the uncertainty expressions pertained, using inductive analysis aimed at generating coding categories from the data.^36^ Second, we assessed whether three conceptually distinguished main sources of uncertainty, that is probability, ambiguity and complexity, and associated subcategories (see Table 2) were reflected in the current data. To that end, we used deductive analysis, originating from predefined codes.^18^, ^37^ Third, all clinicians' uncertainty expressions were categorized based on who initiated or elicited the uncertainty utterance: the clinician, the patient or the caregiver.

## 3 RESULTS

### 3.1 Patient and clinician characteristics

Table 1 displays the characteristics of the 22 clinicians and 78 patients participating. Of these patients, 73 were accompanied by a caregiver, most often their partner (64%). Clinicians each recorded post‐testing consultations with one to eight patients (Mdn = 3) with a mean duration of 18 minutes (SD = 8, range 3‐39 minutes).

**Table 1:** Patient and clinician characteristics

Patients	n = 78
Gender (female)	32 (41%)
Age (in y)	*M* = 70, SD = 11, Range = 43‐91
Mini‐Mental State Examination (MMSE) score	*M* = 25, SD = 4, Range = 12‐30
Diagnostic group	
Dementia	32 (41%)
MCI	13 (17%)
Cognitively normal	19 (24%)
Other/unclear	14 (18%)
Highest level of education	
Primary school/lower level vocational education	23 (30%)
General secondary education	25 (32%)
Higher level vocational/college/university education	18 (23%)
Other	3 (4%)
Tolerance for uncertainty (adapted PRUS)^a^	*M* = 30, SD = 8, Range = 14‐46
Patients accompanied by a caregiver^b^	73 (94%)
A spouse/partner	49 (63%)
A daughter/son (in law)	14 (18%)
Other, for example a sister/brother (in law), niece or friend	5 (6%)
Relationship unknown	5 (6%)
Clinicians	n = 22
Gender (female)	13 (59%)
Age (in y)	*M* = 48, SD = 10, Range = 27‐66
Medical specialty	
Neurologist	14 (64%)
Geriatrician	8 (36%)
Work experience at a memory clinic (in y)	*M* = 10, SD = 7, Range = 0‐25
Number of new patients per month	*M* = 17, SD = 8, Range = 4‐30
Tolerance for uncertainty (PRUS)^c^	*M* = 25, SD = 5, Range = 16‐35

Note

Numbers are n (%), unless otherwise indicated.

a The internal consistency of this scale was acceptable in our sample, with Cronbach's alpha .73

b If the patient was companied by more than one caregiver, we only categorized the main caregiver.

c The internal consistency of this scale was acceptable in our sample, with Cronbach's alpha .72

### 3.2 Uncertainty expressions: descriptives and the relation to characteristics

A total of 115 uncertainties expressions by clinicians were observed, within 57 of the 78 (73%) consultations. Thus, in 21 (27%) consultations the clinician communicated no uncertainties. Clinicians uttered on average 1.7 expressions indicating uncertainty per consultation, ranging from 0 to 7.

**Figure 1:**
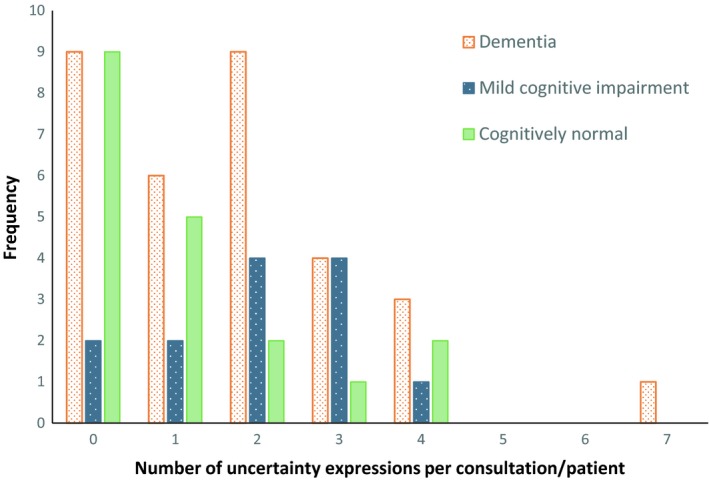
Distributions of the number of uncertainty expressions per consultation/patient, by diagnostic category

We found no relationships between clinician or patient characteristics (all characteristics displayed in Table 1), and whether or how often clinicians communicated uncertainty. An exception was patients' diagnostic category: a trend was found suggesting differences between diagnostic categories in the number of uncertainty expressions uttered by clinicians (Kruskal‐Wallis *H* = 4.84, *P* = .089; Median test Chi‐square = 6.26, *P* = .044). Pairwise comparisons indicated that clinicians expressed uncertainty more frequently when disclosing an MCI diagnosis, compared to a cognitively normal ‘diagnosis’ (Mann‐Whitney *U* = 71.00, *P* = .038). This is visualized in Figure 1.

### 3.3 Uncertainty expressions: topics, causes and elicitation

#### 3.3.1 Topics of uncertainty

Clinicians' uncertainty expressions related to ten different topics (see Table 2, including representative quotes). Most expressions of uncertainty were about (a) patients' diagnosis or the cause of their symptoms, and (b) how their symptoms or disease would develop. In other cases, uncertainty related for example to conflicting test results, the effects of medication or heredity of the disease.

**Table 2:** Topics—stratified by source—and examples

Topic	Source	Frequency	Representative quote
1. Diagnosis: the diagnostic label and/or the cause of the patient's symptoms	Ambiguity	39×	‘It is a working diagnosis. We cannot prove anything, but we think this [diagnosis] fits the profile best.’ (p2112; Male; 70 y; dementia)
	Probability	2×	‘We do not think you have a dementia, but we also don't think your memory is totally fine. It is in sort of an intermediate phase, of which we do not know how it will develop.’ (p6110; Male; 74 y; MCI)
	Complexity	1×	‘Well, it is always difficult to say what causes fatigue.’ (p1112; Female; 65 y; MCI)
2. Course: the course or progress of the disease after receiving a diagnosis	Probability	32×	‘I don't know how this [the symptoms] will develop, nobody knows. Only time can tell.’ (p7106; Male; 73 y; MCI)
	Ambiguity	2×	‘The problem kind of started when you retired. […] The idea is that with that change in your life, you created some shortcomings. So because of that and not because of an underlying disease, some functions are declining. […] And with actively doing things, some functions may improve again. We do not know this for sure, but we think this will be a good start.’ (p2117; Male; 66 y; cognitively normal)
3. Risk: the risk of developing dementia	Probability	8×	‘And again, the fact we do not see any deviations at the moment, does not mean this will still be the case in 10 years.’ (p6109; Female; 65 y; cognitively normal)
	Ambiguity	2×	‘You are scared of becoming demented. Well, we can never tell whether that is happening at a specific moment.’ (p5123; Female; 77 y; other)
4. Test limitations: limitations of the test(ing) material(s)	Ambiguity	8×	‘Based on the scan, I cannot tell if this is normal aging, or too much [shrinkage] for your age.’ (p6103; Female; 69 y; dementia)
5. Medication effects: the effectiveness of available medication	Probability	5×	‘The effects of medication are difficult to predict. Some benefit from it, others don't really.’ (p1106; Male; 73 y; dementia)
	Ambiguity	1×	‘It's a comparison we cannot make, because it is impossible to compare yourself with how you would be without medication.’ (p1106; Male; 73 y; dementia)
6. Test results: (conflicting) test result(s)	Ambiguity	5×	‘We are not completely sure yet. One part of the [neuropsychological] testing went very well, another part didn't. So, there appears to be some disorder based on that. However, the [MRI] scan looks good.’ (p3116; Female; 69 y; MCI)
7. Cause/effect: what is cause and what is effect	Ambiguity	4×	‘It is difficult to say what is cause and what is effect. […] It is possible that your depressed mood is caused by the difficulties you experience.’ (p1105; Female; 64 y; dementia)
8. Disease origin: how/why the disease developed	Ambiguity	3×	‘Caregiver: How do those amyloid plaques form? Clinician: We are still doing a lot of research to figure that out […] I cannot answer this question yet.’ (p1112; Female; 65 y; MCI)
9. Heredity: the heredity of the disease	Probability	2×	‘Patient: So it is not written in stone that Alzheimer's disease is chromosomally inheritable? Clinician: No.’ (p1108; Male; 68 y; dementia)
10. Match brain region and function: what part of the brain is related to what function	Complexity	1×	‘Caregiver: And what [function] is in there [related to that area of the brain]? Clinician: Well, we can't really say. […] For some functions it is possible [to pinpoint a brain region], for instance for language, but for most functions this is not possible.’ (p1110; Female; 66 y; dementia)

#### 3.3.2 Sources of uncertainty

For about half of the uncertainty expressions, the clinician acknowledged his/her inability to predict the future; that is, the uncertainty was due to *probability* (see Table 3, including representative quotes). For example, the clinician addressed the risk of developing dementia (Extract 1), or conveyed uncertainty about predicting the course of the symptoms or underlying disease (Extract 2).

**Table 3:** Sources of uncertainty, their subcategories, descriptions and examples

Source^18^	Description	Representative quote
Probability—no subcategories	Randomness or indeterminacy of future outcomes	**Extract 1**: ‘We know that patients with MCI have a higher risk of developing a dementia. That is about 50% in the coming 3 to 4 years.’ (p1103; Male; 70 y; MCI)
		**Extract 2**: ‘How this will develop and how fast, I don't know either. Nobody knows.’ (p1101; Male; 48 y; dementia)
		**Extract 3**: ‘So we could try this [medication]. The medication does not work for everyone, it works for one out of three people, but that is enough to at least try them.’ (p1109; Female; 58 y; dementia)
Ambiguity—six relevant subcategories:	The lack of reliability, credibility or adequacy of information	
Incompleteness	Insufficiency or inadequacy of information	**Extract 4**: ‘So, we have too little information yet to determine what is going on.’ (p3116; Female; 69 y; [early] dementia)
Indefinitiveness	The lack of a single, precise or invariant answer	**Extract 5**: ‘Of course, we do not have absolute answers to these sorts of questions, because there is something [the matter], otherwise you wouldn't be here. The problem could have started when you retired. […] So an underlying disease is not necessarily the cause of decline. […] By becoming more active, you may experience some improvement. We don't know this for sure, but this could be the cause.’ (p2117; Male; 66 y; cognitively normal)
Tentativeness	The lack of a final, unchanging, definitive answer; undecidedness	**Extract 6**: ‘At this moment I don't see any indications for a brain disease, but I can't completely rule this out.’ (p7104; Male; 72 y; cognitively normal)
Inconsistency	Divergent, dissimilar or contradictory to the norm	**Extract 7**: ‘So we cannot come to a conclusion. Because one part of the testing went very well, and another part didn't. It appears that you clearly have abnormalities [in cognitive functioning], but the MRI is not too bad.’ (p3116; Female; 69 y; dementia)
Polysemousness	Susceptibility to multiple meanings or interpretations	**Extract 8**: ‘What I'm trying to explain is that it is possible we are dealing with someone who had an accident in the past and is now having normal aging in the brain. The effects from the past are more noticeable, because the reserve capacity of the brain is decreasing, because you're getting older. That is one possibility. The other possibility is that we do have to think about a disease of the brain, that is causing early aging of the brain. So, indeed a form of dementia.’ (p6103; Male; 69 y; dementia)
Insolubility	Impossibility of something to be worked out or explained	**Extract 9**: ‘The effect of the medication is difficult to predict. Some experience more benefits than others. What we sometimes notice is that some people become more alert. It is a comparison you cannot make, because you cannot compare yourself with how you were without the medication, that is the whole point.’ (p1106; Male; 73 y; dementia)
Complexity—one relevant subcategory:	Features of information that limit its understanding	
Complexity	Intricateness, multidimensionality or multifacetedness of information	**Extract 10**: Caregiver: ‘What happens over there, in these areas? Or does the brain not work like that? That certain functions are located there?’ Clinician: ‘Well, we can't really say that. For instance, when certain memory structures are really affected, that may say something. But, it is really difficult to predict precisely what kinds of symptoms this results in. For some functions it is possible [to pinpoint the location], for instance for language, but for most functions this does not apply.’ (p1110; Female; 66 y; dementia)

The other half of uncertainty expressions was caused by *ambiguity*, that is related to limits in the quality of information. We could further identify six subtypes of ambiguity, in line with previous conceptual work^29^ (Table 3). Clinicians most frequently expressed uncertainty caused by: (a) limits in (the currently available) knowledge (*incompleteness*), for example when not all test results are yet known (Extract 4); (b) their inability to provide a single solution (*indefinitiveness*), for example the lack of a single diagnosis (Extract 5); or (c) their inability to provide a definitive answer (*tentativeness*), for example about the cause of the patient's symptoms (Extract 6). Other subcategories of ambiguity were found only occasionally; in these cases, the uncertainty was caused by *inconsistency* between test results (Extract 7), the existence of multiple possible meanings, or *polysemousness* (Extract 8) or by the impossibility to systematically test the potential effects of medication, that is *insolubility* (Extract 9).

Complexity was only occasionally the cause of the uncertainty expressed by clinicians in these consultations, for example uncertainty caused by the complexity of disease development and brain functions (Extract 10).

Table 2 illustrates that different uncertainty topics were explained by different sources of uncertainty. Particularly, *probability*, that is the inability to predict the future, was the source of uncertainty in almost all instances in which clinicians expressed uncertainty about disease/symptom progression, the risk of developing a dementia or the effects of medication. For other topics, *ambiguity* was the primary source of uncertainty. For example, when clinicians expressed uncertainty about diagnostic labels or limitations of diagnostic tests, their uncertainty was almost exclusively explained by *ambiguity*.

#### 3.3.3 Elicitation of clinicians' uncertainty expressions

Of the 115 uncertainty utterances expressed by the clinicians, 72 (63%) were initiated by the clinician (Table 4). Of those, in 32/72 (44%), the uncertainty pertained to patients' (working) diagnosis or the cause of their symptoms. Patients and caregivers elicited, respectively, 17/115 (15%) and 26/115 (23%) uncertainty expressions by clinicians, through questions or statements. For example, in one of the consultations, the caregiver remarks that the patient does not show signs of Parkinsonism, and the clinician responds with ‘No, he has not got that at the moment, it is possible that it will develop, but it doesn't have to’. Of note, 17/26 (65%) caregiver‐elicited uncertainty expressions were about the course or progress of the disease/symptoms (eg, ‘You can't say anything about the progression, on how this will proceed?’).

**Table 4:** Topics of clinicians' uncertainty expressions, stratified by initiator: the clinician, the patient or the caregiver

Uncertainty expression about…	Topic initiated/elicited by:			
	Clinician	Patient	Caregiver	Total N
1. Diagnosis: the diagnostic label and/or the cause of the patient's symptoms	32	6	4	42
2. Course: the course or progress of the disease/symptoms	10	7	17	34
3. Risk: the risk of developing a dementia	9	0	1	10
4. Test limitations: limitations of the test(ing) material(s)	7	0	1	8
5. Medication effects: the effectiveness of available medication	4	2	0	6
6. Test results: (conflicting) test result(s)	6	0	0	6
7. Cause/effect: what is cause and what is effect	3	0	0	3
8. Disease origin: how/why the disease developed	1	0	2	3
9. Heredity: the heredity of the disease	0	2	0	2
10. Match brain and function: what part of the brain is related to what function	0	0	1	1
Total N	72	17	26	115

## 4 DISCUSSION AND CONCLUSION

### 4.1 Discussion

This is the first study examining whether and to what extent clinicians discuss with patients the uncertainty associated with results of diagnostic testing at a memory clinic. Discussion of uncertainty is prevalent in these memory clinic consultations; in three‐quarter of the consultations, uncertainty was addressed at least once. These uncertainties pertained to various topics, such as diagnosis, disease progression and heredity. Roughly half of them were related to clinicians' inability to predict the future (‘probability’) and the other half to limitations (eg, incompleteness, unreliability) to the knowledge available (‘ambiguity’). This means that, if clinicians expressed uncertainty, they emphasized not only their inability to predict symptom and disease progression, but also the limits to scientific knowledge and to the reliability of diagnostic tests and test results. In contrast, previous analyses of oncological consultations indicated that clinicians did not as openly discuss the limits of scientific knowledge.^38^ Their hesitance was caused by fear that such information would be too complex or cause anxiety in patients.^39^, ^40^ It is therefore surprising that in the present study, many clinicians were more outspoken about the ambiguity surrounding diagnosis and testing. This may be because the present consultations had a diagnostic purpose, whereas research in the oncology setting involved more discussion about treatment. When decisions about potentially life‐saving treatments need to be made, openly discussing the limits of evidence may complicate treatment decision making by inducing doubt in patients.^41^ This is different in AD, where treatment options as of yet are extremely limited. Open communication about uncertainties may feel normal or unavoidable to memory clinic clinicians, even in case of a more ‘certain’ diagnosis like dementia. Clinicians apparently wanted to make patients aware that such a certain diagnosis still entails uncertainty about, for example, the progression of symptoms or the effectiveness of available medication.

How clinicians' open acknowledgement of their uncertainties affects patients has not been investigated. Once informed, patients have to deal with these uncertainties. This may be difficult for patients, as illustrated by our finding that clinicians' uncertainty expressions were frequently in response to patients' and caregivers' requests for more certainty about disease progression or symptoms. Future research should establish whether the advantages of early testing outweigh the disadvantage of having to deal with uncertainty.^42^ If possible, patients should be given the choice between whether or not they want to undergo early diagnostic testing.^43^, ^44^ To facilitate that choice and help them manage their expectations, they need to be aware of possible uncertainties prior to testing.^45^ A recent Delphi consensus study by our group indicated that clinicians, patients and caregivers agree about the importance of discussing the potentially uncertain result of diagnostic testing during pre‐testing clinician‐patient consultations.^46^

Noticeably, in about one quarter of disclosure consultations, clinicians did not address uncertainty at all. We do not know if this is worrisome, because there is no evidence of a clear net benefit or disadvantage of communicating uncertainty. At the one hand, most patients prefer to be made aware about uncertainty,^32^ and such awareness, for example about the limitations of test results, enables them to more adequately interpret the results and cope with diagnosis.^47^, ^48^ Moreover clinicians' disclosure of uncertainty may overall strengthen patients' perception of clinicians' honesty and respectful attitude, which may enhance trust.^49^, ^50^ Awareness of uncertainty can even function as a source of hope for patients, by leaving open the possibility that negative outcomes (eg, developing dementia) might/may *not* occur.^22^, ^51^ Clinicians might thus in some cases purposefully express uncertainty to help patients cope with their situation.^52^, ^53^ On the other hand, it might not always be warranted to communicate uncertainty. For example, based on our explorative analyses, clinicians may express uncertainties less frequently in consultations with cognitively normal individuals, potentially because the test results and their implications for the future are more clear‐cut in those cases than, for example, in individuals labelled with MCI. Furthermore, among patients, high levels of uncertainty have been related to increased worry, emotional distress, anxiety and depression.^54^, ^55^, ^56^, ^57^, ^58^ To avoid harming patients, clinicians may need to not only inform patients about uncertainty, but to additionally support them in dealing with it.^59^ A useful three‐step approach to provide support has been recently proposed.^60^ First, clinicians can normalize uncertainty, by acknowledging patients' wish for more certainty while explaining that uncertainty is unfortunately inherent to the situation. Second, they can acknowledge and address patients' and caregivers' emotions regarding uncertainty by acknowledging that it is unpleasant not to know things. Third, they can help patients and caregivers cope with uncertainty by stimulating them to focus on living in the here and now instead of dwelling on the uncertainty. The effectiveness of these and other proposed strategies remains to be investigated.

### 4.2 Strengths and limitations

Among the strengths of this study are its multicentre design, in which we sampled 78 consultations at eight different Dutch memory clinics. Moreover, we were able to gain rich insight into the data by combining qualitative with quantitative methods. Some limitations deserve mentioning. First, our modest sample size limited statistical power to detect small or moderate effects regarding the moderating influence of patient and clinician characteristics and prevented us from taking into account the hierarchical data structure. Besides one trend, our current exploration did, therefore, not identify promising avenues for further investigation. Thus, future studies should look into individual differences and moderating factors, such as whether the patient's diagnosis or disease stage influences the discussion of uncertainty. Clinicians' tolerance for uncertainty may also be an interesting characteristic to examine more thoroughly. Results from a recent survey study indicate that clinicians who are less tolerant for uncertainty prefer a more paternalistic approach in medical decision making.^61^ If clinicians' tolerance for uncertainty is an important factor for the extent to which they share uncertain information with patients, interventions aimed at enhancing tolerance for uncertainty may eventually contribute to a more adequate discussion of uncertainty. Second, response bias might be a limitation in that sense that clinicians who are more comfortable with communication, including communication of uncertainty, may have been more likely to participate in this study.

### 4.3 Future research directions

First, future research should assess which motives underlie some clinicians' hesitance to express uncertainty. For example, clinicians may not want to add to the large amount and complexity of information they share with patients out of a fear of cognitive overload,^62^ especially in this population. Cognitive overload from too much information could impair recall of information and understanding, thereby possibly evoking unwarranted uncertainty and anxiety in patients and their caregivers. Next, we thus need research to establish how clinicians can optimally communicate uncertainty to patients, by establishing what the effects of different communication strategies are on patients' understanding of the provided information, experienced feelings of anxiety, uncertainty, hope and coping behaviours. Moreover, we should test whether patients benefit from being informed about the possible uncertainties *prior* to testing. Third, we should investigate how we can facilitate clinicians to optimally communicate uncertainties to patients. We simultaneously need to establish how clinicians may balance such an approach with tailoring their information to patients' individual needs. To investigate these questions, both quantitative and qualitative measures should be employed. The current investigation provides a useful starting point to establish insight into uncertainty discussion in the memory clinic setting. Future studies need to go beyond quantification to develop a richer understanding of how uncertainty communication shapes the clinician‐patient interaction and how such communication is shaped by the characteristics of its participants. To expand on our current approach using interpretative content analysis, future research could adopt a linguistic coding method, ascertaining the presence of uncertainty expressions based on strict linguistic criteria.

## 5 CONCLUSION

In addition to communicating their inability to predict symptom and disease progression, many clinicians openly communicated the limits of scientific knowledge and diagnostic testing with patients. However, in approximately one quarter of consultations concerning the disclosure of results of diagnostic testing in the context of dementia no uncertainties were communicated. More research is warranted to investigate the beneficial and/or harmful impact of uncertainty communication, to establish how clinicians can optimally discuss uncertainty with patients, that is in a way that would benefit and not harm patients. Eventually, such research can result in useful advice and/or communication tools for memory clinic clinicians, thereby improving the quality of care provided to patients and their caregivers.

## CONFLICT OF INTEREST

Leonie NC Visser, Sophie AR Pelt, Marleen Kunneman, Femke H. Bouwman, Jules J. Claus, Kees J. Kalisvaart, Liesbeth Hempenius, Marlijn H. de Beer, Gerwin Roks, Leo Boelaarts, Mariska Kleijer, Ellen MA Smets, and Marij A. Hillen declare no conflict of interests. Prof. Dr. Wiesje M. van der Flier performs contracted research for Biogen MA Inc. Her research programmes have been funded by ZonMW, Health Holland, Pasman stichting, NWO, EU‐FP7, EU‐JPND, Alzheimer Nederland, Cardiovasculair Onderzoek Nederland, stichting Dioraphte, Gieskes‐Strijbis fonds, Boehringer Ingelheim, Piramal Neuroimaging, Roche BV, Janssen Stellar, and Combinostics. All funding is paid to the institution.

## Data Availability

Research data are not shared (for privacy reasons).
